# Determination of Cadmium, Copper, Lead, and Silicon in Anti-Hair Loss Dietary Supplements That Contain Various Plant-Derived Silicon Sources

**DOI:** 10.3390/foods15193577

**Published:** 2026-10-08

**Authors:** Magdalena Krawczyk-Coda

**Affiliations:** Faculty of Chemical Technology, Poznan University of Technology, Berdychowo 4, 60-965 Poznań, Poland; magdalena.krawczyk@put.poznan.pl

**Keywords:** high-resolution continuum source GFAAS, cadmium, copper, lead, silicon, anti-hair loss dietary supplements

## Abstract

In this research, an accurate procedure was developed to determine Cd, Cu, Pb and Si in dietary supplements using high-resolution continuum source graphite furnace atomic absorption spectrometry (HR-CS GFAAS). The operational parameters for the graphite furnace method were thoroughly investigated, including the influence of the chemical modifier along with the optimisation of pyrolysis and atomisation temperatures. The detection limits for Cd, Cu, Pb, and Si were 0.02 µg L^−1^, 0.35 µg L^−1^, 0.18 µg L ^−1^ and 0.98 µg L ^−1^, respectively. The accuracy of the method was validated using INCT-MP4-2 (Mixed Polish Herbs) as a certified reference material and complemented by standard addition tests. The determined elemental contents showed satisfactory agreement with both certified values and the spiked amounts, as confirmed by a *t*-test at 95% confidence level. The validated procedure was successfully applied to nine commercial dietary supplements representing three botanical silicon sources (bamboo, horsetail, and nettle). The analysis revealed a cadmium concentration in one horsetail-based sample (H1) that marginally exceeded the EU regulatory limit, as well as a substantial discrepancy between the declared and measured silicon content in another horsetail-based sample (H2), where the determined value was nearly eight times lower than stated by the manufacturer.

## 1. Introduction

Globally, hair loss cases have seen a significant increase in recent years, with androgenetic alopecia (AGA) projected to affect approximately 80% of men and 50% of women during their lifetimes [1]. From a dermatological standpoint, this condition arises when the biological hair growth cycle is disrupted or when follicles suffer structural damage. The literature suggests that the underlying triggers represent a multifaceted mix of modern lifestyle shifts, elevated stress, environmental pollution, genetic predisposition, nutritional deficiencies, certain medications, and hormonal imbalances, including disorders such as hypothyroidism, hyperthyroidism, and polycystic ovary syndrome [2]. While successful hair loss intervention in clinical practice relies strictly on diagnosing the underlying medical etiology, management often incorporates pharmacological therapies and dietary adjustments.

As the second most prevalent element on Earth, silicon also ranks as the third most abundant trace element in the human body. Obtainable from water, plant and animal sources, silicon has been reported to play a role in hair physiology; some studies suggest that elevated concentrations of silicon in hair fibres are associated with reduced shedding and enhanced lustre [3]. Similarly, copper has been linked to hair vitality due to its role in haemoglobin synthesis, a process required to deliver oxygen to the hair shafts, and copper deficiency has been reported to compromise the integrity of the shaft and contribute to hair loss [4]. It should be emphasised that these associations originate from the existing clinical and nutritional literature and are not addressed or verified by the present study, which is limited to the determination of the concentrations of selected elements.

In the literature, diet supplements fortified with biotin, essential vitamins, and botanical silicon sources such as bamboo, horsetail, and nettle have been proposed as a means of supporting hair health, particularly when combined with a nutritionally balanced diet [5]. However, such reported benefits pertain to nutritional and clinical outcomes rather than elemental content per se, and the present study does not evaluate clinical efficacy, hair growth parameters, bioavailability, or nutritional status. Plant-derived dietary supplements often contain trace amounts of cadmium and lead, as vegetation naturally takes up these heavy metals from the surrounding soil and the Earth’s crust. Beyond the inherent contamination of raw botanical materials, finished products can undergo further contamination due to unintentional cross-contamination during manufacturing or by chemical migration from product packaging [6].

Driven by the growing market for these formulations, ensuring consumer safety through rigorous quality control has become an paramount analytical task. The evaluation of plant-derived dietary supplements requires the use of robust analytical methodologies to verify identity, quantify active constituents, and detect potential contamination [7]. Globally accepted techniques for quantifying Cd, Cu, Pb, and Si levels in complex matrices include flame atomic absorption spectrometry (FAAS) [5,8,9], graphite furnace atomic absorption spectrometry (GFAAS) [10,11], inductively coupled plasma optical emission spectroscopy (ICP-OES) [12,13], or inductively coupled plasma mass spectrometry (ICP-MS) [14,15].

Although the majority of the published analytical literature focuses on determining silicon or copper separately—or toxic metals independently—there is a lack of widely accessible procedures capable of simultaneous or sequential determination of all four elements within a single sample preparation framework. The aim of this study is strictly methodological: to develop and validate a widely accessible analytical procedure capable of determining silicon, copper, lead, and cadmium in commercial botanical dietary supplements. Although most previous studies have evaluated supplements containing silicon derived from only one or two distinct botanical sources, this work offers a broader analytical screen across multiple sources. To our knowledge, this is the most comprehensive quality control survey to date that evaluates dietary supplements incorporating silicon derived from three different botanical sources: bamboo, horsetail, and nettle extracts. This research addresses a critical aspect of analytical quality control, which is essential because these specific hyperaccumulating plants pose a high risk of heavy metal contamination, requiring stringent verification independent of any therapeutic claims.

## 2. Materials and Methods

### 2.1. Reagents

Ultra-high-purity (UHP 5.5) compressed argon, supplied by Air Products (Warsaw, Poland), served as the inert gas and was used without additional purification. Cd, Cu, Pb, and Si working standard solutions were prepared by appropriately diluting commercially available 1000 mg L^−1^ atomic absorption stock standards (Merck, Darmstadt, Germany).

Stock modifier solutions of palladium (II) nitrate (Pd(NO_3_)_2_) and magnesium nitrate (Mg(NO_3_)_2_), both at a concentration of 10.0 ± 0.2 g L^−1^, were also purchased from Merck. Sample digestion was performed using high-purity reagents from Merck’s Suprapur line: 65% (*v*/*v*) nitric acid, 40% (*v*/*v*) hydrofluoric acid, and 30% (*v*/*v*) hydrogen peroxide. Ultrapure water (18 MΩ·cm resistivity), generated using a Bi18 quartz distillation unit (Heraeus, Hanau, Germany), was used throughout all experiments.

### 2.2. Certified Reference Material

The certified reference material INCT-MP4-2 (Mixed Polish Herbs), provided by the Institute of Nuclear Chemistry and Technology in Warsaw, Poland, was used to verify the accuracy of the proposed analytical method. The techniques used most frequently for the determination of Cd, Cu, and Pb were atomic absorption spectrometry (AAS), inductively coupled plasma atomic emission spectrometry (ICP-AES) and inductively coupled plasma mass spectrometry (ICP-MS) [16].

### 2.3. Anti-Hair Loss Dietary Supplements

This study evaluated nine anti-hair loss dietary supplements formulated with plant-derived silicon sources, specifically bamboo (B1–B3), horsetail (H1–H3), and nettle (N1–N3). All products were sourced from a local pharmacy, purchasing five separate packages per supplement from different batches of production. During the research, nine different products were analysed. Each supplement was produced by a different manufacturer. All nettle-sourced silicon supplements were formulated as tablets, while those utilising bamboo or horsetail were formulated as capsules. Before digestion, homogeneity of each sample was achieved by manual grinding in a ceramic mortar, subsequent thorough mixing, and sieving through a 2 mm sieve.

### 2.4. Microwave-Assisted High Pressure Teflon Bomb Digestion

The digestion of the samples was carried out with a UniClever focused microwave system (Plazmatronika, Wrocław, Poland), operating at a frequency of 2450 MHz and a maximum power output of 300 W. The instrument’s computer-controlled interface allowed real-time monitoring of temperature, pressure, and microwave power throughout the procedure. Digestion was carried out in 110 mL high-pressure TFM-PTFE vessels capable of withstand up to 100 atm and 300 °C.

Approximately 350 mg of the powdered supplement was weighed in a TFM-PTFE vessel using an M2P microanalytical balance (Sartorius, Göttingen, Germany) and pre-wetted with 1 mL of 30% H_2_O_2_. Subsequently, 5 mL of 65% HNO_3_ and 0.3 mL of 40% HF were added. The mixture was irradiated in the microwave system for 20 min at 300 W. Following digestion, the vessels were opened under a fume hood, and the samples were evaporated on a heating block at 130 °C until the volume was reduced to approximately 1 mL. To prevent losses of volatile analytes, the temperature was strictly controlled, and the solutions were not allowed to evaporate to dryness. The resulting clear solutions were quantitatively transferred into 25 mL volumetric flasks and diluted to volume with high-purity water. The optimization of the digestion procedure revealed that as little as 0.3 mL of 40% HF was sufficient for total matrix decomposition; moreover, maintaining such a low acid volume effectively mitigated its corrosive effects, rendering the digested solutions perfectly suitable for direct HR-CS GFAAS analysis. Since silicon reacts with the carbon of the graphite tube at 2600 °C to form stable, non-volatile silicon carbide (SiC)—which causes severe memory effects and tube degradation—the utilization of a pyrolytically coated graphite tube in this study effectively prevented SiC formation, ensuring stable and reproducible analytical signals [17]. Prior to analysis, further dilutions were performed as required, depending on the concentration levels of Cd, Cu, Pb, and Si. Blank solutions were prepared concurrently using the identical microwave-assisted digestion procedure.

### 2.5. AAS Determination Procedure

All measurements were carried out on a high-resolution continuum source atomic absorption spectrometer fitted with a 300 W xenon short-arc lamp (ContrAA 700, Analytik Jena, Jena, Germany). A graphite furnace containing a pyrolytically coated, platform-equipped graphite tube served as the atomisation source. The introduction of the sample (20 µL) and the addition of modifier (5 µL) to the graphite tube were performed automatically using an autosampler. Following the measurements, the data stored in the instrument’s internal memory were transferred to a computer for analysis. Peak height and peak area signals were recorded following signal integration. Since the precision of the peak height measurements outperformed that of the peak area, the former was chosen for all subsequent evaluations. To compensate for any potential contamination introduced by the sample preparation reagents, analytical blanks were carried through the entire procedure. After all calculations, the blank value was subtracted from the sample results. Linear calibration curves were utilised for the quantification of Cd, Cu, Pb, and Si. The operating parameters of the HR-CS GFAAS instrument are summarised in Table 1.

## 3. Results and Discussion

### 3.1. Selection of Instrumental Conditions

The xenon short-arc lamp was operated at a current of 9 A. To maximise sensitivity, the primary analytical lines selected for quantification were 228.802 nm for Cd, 324.754 nm for Cu and 251.611 nm for Si. Since the analysed supplements feature a complex botanical matrix co-formulated with heavy structural excipients, the resulting digested solutions contain elevated concentrations of concomitant elements. During HR-CS GFAAS determination, matrix components such as phosphorus (P), calcium (Ca), and iron (Fe) introduce substantial chemical interferences as well as molecular background absorption. Calcium, originating from plant accumulation and dicalcium phosphate fillers, forms stable gaseous oxide and hydroxide molecules in the atomisation cell. These molecules generate broadband absorption profiles that can shift or suppress Cu and Pb atomisation peaks [18]. Phosphorus originates from botanical nucleic acids, cellular phospholipids, and phosphate-based excipients. Using the most sensitive wavelength for Pb results in spectral interferences; specifically, the atomic absorption line at 217.001 nm overlaps with PO molecular absorption bands. Consequently, Pb was determined using a less sensitive alternative wavelength at 283.306 nm. Iron exhibits a line-rich atomic absorption spectrum, and its lines located near the Cu or Pb analytical lines can induce direct atomic spectral overlap.

To successfully mitigate and prevent these interferences, a combination of chemical, instrumental, and tailored calibration strategies must be used. Modifiers are widely used in HR-CS GFAAS to stabilise volatile analytes, enabling higher pyrolysis temperatures to eliminate matrix components before atomisation. This high-temperature pyrolysis substantially decreases spectral and chemical interferences, while a suitable modifier influences sample–graphite interactions and standardises the atomisation of different analyte species [19,20].

In this work, the chemical modifier was applied as a mixture of Pd(NO_3_)_2_ (1.5 μg μL^−1^) and Mg(NO_3_)_2_ (1.0 μg μL^−1^). The application of a 5 µL modifier volume increased the analytical signals for the target elements by approximately 25–30% while producing well-resolved, reproducible peaks. Because larger modifier volumes (>5 µL) did not provide any additional improvement to the analytical signals, an optimal volume of 5 µL was chosen for all subsequent analyses.

**Figure 1:**
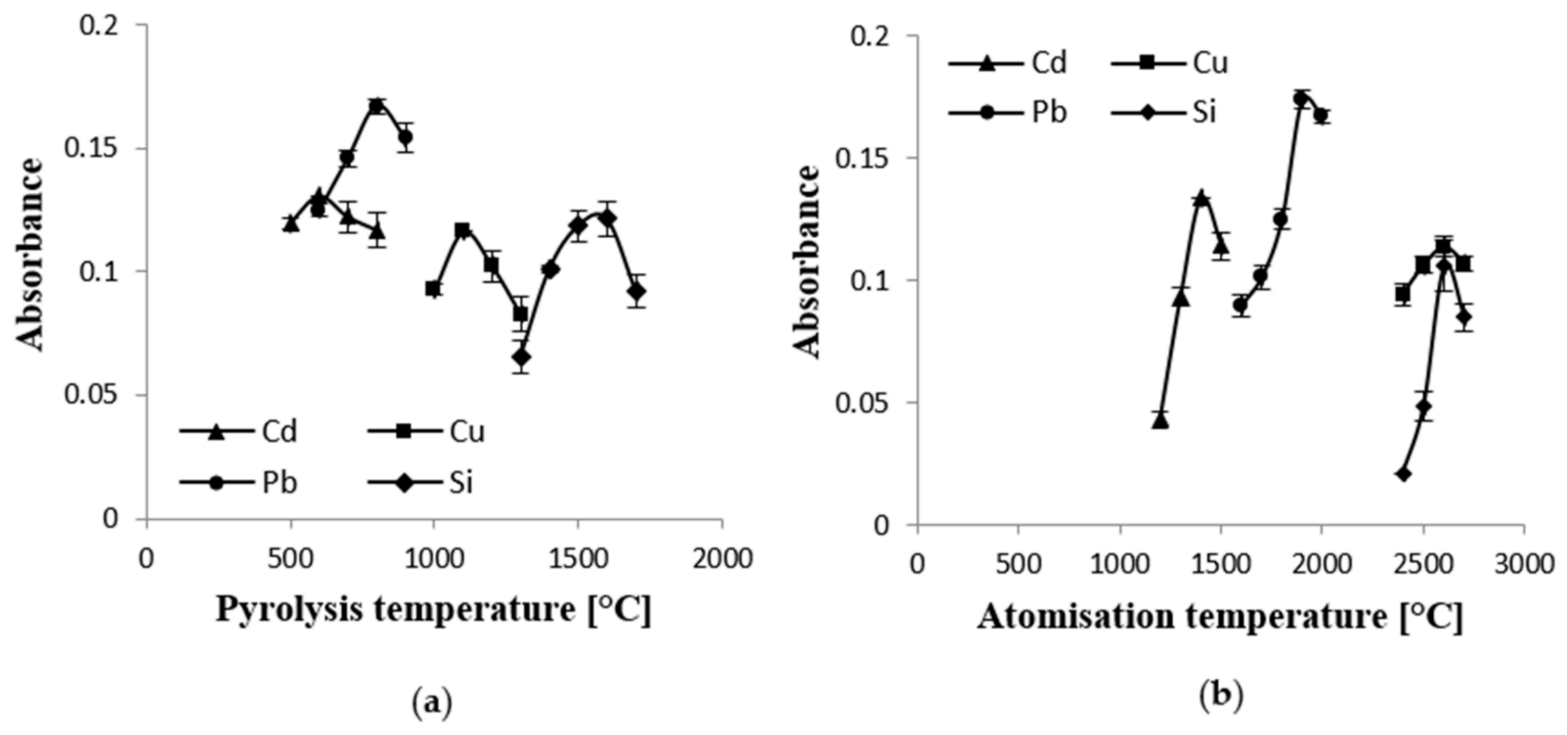
Optimisation of the temperature programme: (**a**) Pyrolysis temperature. (**b**) Atomisation temperature. The error bar is the standard deviation (*n* = 5).

The optimisation of the temperature programme was performed using a mixed standard solution of Cd (1 μg L^−1^), Cu (5 μg L^−1^), Pb (15 μg L^−1^) and Si (25 μg L^−1^). To avoid sample spattering and promote homogeneous solid deposition on the graphite surface, a three-step drying sequence was consistently applied. In HR-CS GFAAS analysis, the pyrolysis temperature represents the most pivotal variable in the heating cycle. Consequently, its influence on absorbance was evaluated in the ranges of 400 to 800 °C (Cd), 1000 to 1300 °C (Cu), 600 to 900 °C (Pb) and 1300 to 1700 °C (Si) using a mixture of Pd(NO3)2 and Mg(NO3)2 as a modifier (Figure 1a). Optimal pyrolysis temperatures that produce the maximum absorbance were found to be 600 °C, 1200 °C, 800 °C, and 1600 °C for Cd, Cu, Pb, and Si, respectively.

Following optimisation of pyrolysis conditions, the effect of atomisation temperature on analytical signals was investigated within the ranges of 1200–1500 °C (Cd), 2400–2700 °C (Cu), 1600–2700 ° C (Pb) and 2400–2700 °C (Si) (Figure 1b). For this evaluation, the optimum pyrolysis temperatures determined previously for each element were applied. Maximum absorbance was achieved at atomisation temperatures of 1400 °C, 2500 °C, 1900 °C, and 2600 °C for Cd, Cu, Pb, and Si, respectively. Complete vaporisation and atomisation of the target elements were achieved in the graphite tube, with minimal matrix interference. The complete temperature programmes utilised for the determination of the selected elements in the certified reference material and dietary supplements are shown in Table 1.

### 3.2. Analytical Figures of Merit

The LOD and LOQ values were established following the recommendations of IUPAC, using the equations LOD = 3 SD/m and LOQ = 10 SD/m, where SD represents the standard deviation of ten successive blank measurements and m is the slope derived from the calibration curve [21]. This approach yielded LOD values of 0.02 µg L^−1^ (Cd), 0.35 µg L^−1^ (Cu), 0.18 µg L^−1^ (Pb), and 0.98 µg L^−1^ (Si), with corresponding LOQ values of 0.06, 1.16, 0.60, and 3.25 µg L^−1^, respectively.

Although a direct comparison of detection limits can be misleading due to the varying instrumentation, operating conditions, and sample preparation modes, the LODs obtained in this study are highly comparable to those reported in the literature. Specifically, Maziero et al. reported LODs for Cd (0.02 µg L^−1^), Cu (11 µg L^−1^), and Pb (0.27 µg L^−1^) [22], while Shaltout et al. achieved values of 0.03 µg L^−1^ for Cd, 0.49 µg L^−1^ for Cu, and 0.59 µg ^−1^ for Pb using HR-CS GFAAS [23]. Furthermore, the LOD achieved for silicon in the present work was nearly eighty times lower than the value (0.08 mg L^−1^) reported by Pires et al. [24] and three times lower than that (3 µg L ^−1^) presented by Dravecz et al. [25].

The precision of the method was demonstrated by values of relative standard deviation (RSD) spanning 3.8–4.4% (Cd), 2.3–5.1% (Cu), 1.6–4.3% (Pb), and 4.1–5.6% (Si), reflecting highly reproducible results across the entire analytical procedure. The analytical performance of the developed method was evaluated using seven-point external calibration curves, generated at the start of every analytical sequence. The calibration curves displayed strong linearity within the working concentration ranges of 0.25–5 µg L^−1^, 2.5–25 µg L^−1^, 2.5–50 µg L^−1^, and 10–100 µg L^−1^ for Cd, Cu, Pb, and Si, respectively. The representative calibration equations and their corresponding correlation coefficients were as follows: y = 0.069x + 0.0105 (R^2^ = 0.9983) for Cd; y = 0.018x + 0.0107 (R^2^ = 0.9990) for Cu; y = 0.0046x + 0.0027 (R^2^ = 0.9991) for Pb; and y = 0.0029x − 0.002 (R^2^ = 0.9999) for Si. To ensure the statistical validity of the calibration models, strict acceptance criteria were applied: the correlation coefficients were required to be ≥0.995, and the residual back-calculated concentrations for individual standard points had to fall within ±10–15% of their nominal values. Furthermore, system stability and the absence of instrumental drift were monitored throughout the runs using continuing calibration verification (CCV) standards, with an acceptable recovery threshold established at 90–110% of the expected concentration.

### 3.3. Evaluation of the Environmental Impact

To assess the environmental sustainability of the analytical procedure developed, the open-access AGREEprep tool (v1.0, developed by Wojnowski et al., available via MOST Wiedzy) was used, evaluating the sample preparation workflow in accordance with the 10 principles of green sample preparation [26]. The procedure yielded a final greenness score of 0.46, representing a balanced trade-off between environmental sustainability and analytical rigour. Criteria requiring significant improvement are: hazardous materials (score 0.07, weight 5), sample throughput (score 0.26, weight 3), and sample preparation placement ex situ (score 0.00, weight 1). Although the use of hazardous acids HNO_3_ and HF heavily penalised the final score, it remains an analytical necessity. Because silicon embedded in plant matrices is highly resistant to dissolution, hydrofluoric acid is indispensable to ensure complete dissolution and accurate total elemental determination. The environmental profile of the method could be optimised by replacing single-position equipment with an automated, multi-vessel mineralisation system. This transition allows for high-throughput batch processing, which substantially decreases both the total analysis time and the energy footprint calculated per sample, directly addressing the core limitations highlighted by the AGREEprep evaluation. In addition, other important criteria, such as sample volume or waste production, can also be improved. Criteria that are already satisfactory and do not require significant improvement are energy consumption, integration and automation, post-sample preparation configuration for analysis, operator’s safety, and sustainability, renewability, and reusability of materials.

### 3.4. Accuracy Verification

To validate the accuracy and precision of the developed method, the INCT-MP4-2 certified reference material (CRM) (mixed Polish Herbs) was analysed. This specific material was selected due to the commercial unavailability of certified reference materials for dietary supplements. It provides certified values for nearly all target analytes, with the exception of Si, which was validated using the standard addition method. The analytical results for CRM are summarised in Table 2. The repeatability of the method was quantified as the standard deviation (SD) across five replicate measurements. A Student’s *t*-test at a 95% confidence level confirmed close agreement between the obtained results and the certified values, demonstrating the suitability of the developed method for determining Cd, Cu, Pb, and Si in complex plant matrices after digestion.

### 3.5. Standard Addition Tests

To further confirm the accuracy of the developed method, selected dietary supplement samples were spiked with known amounts of Cd, Cu, Pb, and Si and subjected to standard addition tests. As shown in Table 3, the results obtained agreed closely with the spiked concentrations and a Student’s *t*-test at 95% confidence level revealed no statistically significant differences. These outcomes confirm that the proposed method is robust and reliable for determining Cd, Cu, Pb, and Si in complex dietary supplement matrices.

### 3.6. Determination of Cd, Cu, Pb, and Si in Dietary Supplements

The concentration of silicon and copper in plant-derived dietary supplements is highly variable, depending on the composition of the raw material, manufacturing practices, and the final formulation. Botanical sources differ drastically in their ability to absorb trace elements from the soil. The absorption of Cu and Si from the roots is heavily influenced by soil pH, synthetic fertilisers, and geographical location. Furthermore, there is a natural balance between these elements; high baseline Si can restrict the bioavailability and distribution of Cu within tissues, protecting the plant from heavy metal stress [27].

Manufacturing protocols further alter elemental yields. Extracting bioactives with different solvents shifts mineral profiles, as seen with water-soluble orthosilicic acid. Additionally, crude milling versus advanced standardisation techniques modifies the final elemental ratios. Processing additives such as binders, flow agents, and stabilisers also modulate mineral levels, while inadequate raw material filtration or mineral-based processing aids can accidentally introduce or inflate trace copper concentrations.

Finally, the physical delivery format affects the final concentrations and elemental interactions. Tablet production requires heavy mechanical compression and large volumes of excipients or synthetic silicates, which can dilute the plant matrix and skew the total Si calculations. In contrast, capsules typically contain loose, dehydrated extracts with fewer additives, allowing the botanical mineral profile to remain closer to its native state [28].

To evaluate the practical applicability of this optimised method for the simultaneous determination of all four elements in real dietary supplements, the samples were analysed under the experimental conditions established. The corresponding results are shown in Table 4. An external standard calibration was used to quantify the selected elements.

Unlike earlier reports that restrict their scope to one or two silicon sources, our approach offers a wider analytical angle, providing, to our knowledge, one of the most comprehensive evaluations of dietary supplements combining silicon from three separate botanical origins—bamboo, horsetail, and nettle. By investigating these specific formulations, this work contributes to addressing a quality assurance gap. Given that these plants are well-documented for their strong capacity to uptake and bioaccumulate toxic elements from the soil, rigorous monitoring of their safety profiles remains essential to mitigate potential product contamination risks. The practical necessity of such monitoring is further illustrated by the analytical results obtained for the target elements in this study.

The experimental data reveal that the maximum concentration of cadmium occurred in sample H1 of the dietary supplement, which is formulated with horsetail extract. According to the Regulation (EU) 2021/1323 of the Commission of 10 August 2021, the maximum permissible cadmium threshold for dietary supplements is restricted to 1.0 µg g ^−1^ [29]. This statutory limit was marginally exceeded by sample H1. Furthermore, sample B2—containing bamboo extract—displayed an elevated level of cadmium that closely approached the legal threshold. Cadmium contamination can originate from several sources. In addition to the natural contamination found in raw botanical ingredients, final products can undergo further contamination during the manufacturing process. This can occur due to accidental cross-contamination on the production line or through the migration of chemicals from the product packaging [30]. The cadmium concentrations in all remaining samples were substantially below the legally allowed limit.

Regarding lead contamination, data indicate that concentrations peaked in sample B1 of the dietary supplement containing bamboo extract, while minimum levels were recorded in samples H1 and H3 containing horsetail extract. The statutory threshold for the content of lead in dietary supplements is set by the Commission Regulation (EU) 2021/1317 of 9 August 2021 [31]. All analysed samples strictly complied with the regulation, which limits the maximum allowed concentration of lead to 3.0 µg g^−1^.

Beyond toxicological safety, essential trace elements also exhibited different distribution patterns. Among the nine supplements examined, bamboo-derived samples showed comparatively high copper content, while nettle-derived samples showed comparatively low copper content. However, given the limited number of products analysed per botanical source (*n* = 3), these observations should be regarded descriptive trends rather than statistically validated group differences. In particular, considerable variability between products in copper concentration was observed within the horsetail group, with sample H2 exhibiting a markedly higher copper content than the other horsetail-derived samples (H1, H3). This heterogeneity likely reflects differences in raw material sourcing, growth conditions, or manufacturing practices between individual products, rather than a characteristic feature of horsetail-based supplements as a group. Horsetail is a highly resilient species characterised by a distinct ability to uptake and accumulate metals directly from the surrounding substrate, and soil conditions at the growth site, including pH levels, have been reported to significantly affect copper accumulation in this plant [32]. The manufacturers declared the copper content per tablet (or capsule) for only four of the analysed samples: B1 (250 µg), B2 (250 µg), B3 (500 µg), and H2 (500 µg). The contents determined during this study were 179.7 ± 2.1 µg (B1), 213.3 ± 3.6 µg (B2), 505.3 ± 21.2 µg (B3), and 514.4 ± 25.8 µg (H2).

Finally, the results demonstrate that the supplements containing bamboo extract yield the highest silicon content. The accumulation of silicon in bamboo varies between species and tissue types, reaching up to 183 mg g ^−1^ of dry matter in leaves, stems, and young shoots alike. Although the literature generally prioritises shoot-derived extracts as richer in silicon compared to leaves, the actual content is heavily influenced by rhizome morphology and genotypic variation among species [33]. Studies confirm that the horsetail serves as another potential source of silicon. Published data indicate that dried horsetail produces a total silicon concentration between 21.11 mg g ^−1^ and 32.80 mg g ^−1^ (accounting for approximately 2.1% to 3.3% elemental silicon). On a broader scale, botanical estimates project the overall silica and silicic acid content to fall between 5% and 10% [34]. The lowest silicon levels were observed in supplements containing nettle extract, which corresponds with literature data identifying nettle as having the poorest concentration of this element. Nettle dry matter contains 1% to 4% natural silica [35]. Manufacturers specified the silicon content per tablet or capsule for only four analyzed samples: B1 (5000 µg), B2 (5000 µg), B3 (5000 µg), and H2 (2500 µg). The levels quantified in this study were 5231 ± 207 µg (B1), 4449 ± 150 µg (B2), 4150 ± 165 µg (B3), and 319.5 ± 12.6 µg (H2). While the results for samples B1B3 align closely with the manufacturers’ claims, the silicon content in sample H2 is nearly eight times lower than declared.

To determine whether the botanical origin of the dietary supplements introduces statistically significant variations in element concentrations, a one-way ANOVA was performed on the mean values of the three plant groups (bamboo, horsetail, and nettle). Statistical analysis did not reveal significant differences between the groups for any of the elements tested, producing specific test statistics and probability values of *F* = 0.5438, *p* = 0.6067 for Cd; *F* = 1.3859, *p* = 0.3200 for Cu; *F* = 1.9966, *p* = 0.2164 for Pb; and *F* = 1.3055, *p* = 0.3383 for Si. These high *p*-values (*p* > 0.05) indicate that the null hypothesis cannot be rejected and that the substantial variations observed in individual samples—such as the elevated copper content in sample H2 or the prominent silicon level in B1—reflect distinct product formulations, raw material source, and specific manufacturing processes rather than uniform chemical features inherent to botanical groups as a whole.

## 4. Conclusions

The results obtained demonstrate that the developed HR-CS GFAAS method is highly robust and suitable for the determination of Cd, Cu, Pb and Si in plant-based dietary supplements.

From a nutritional perspective, the findings indicate that dietary supplements formulated with bamboo extract exhibited higher measured Cu and Si concentrations within the set of investigated samples. However, the alarming discrepancy discovered in sample H2—where the actual silicon content was nearly eight times lower than the manufacturer’s declaration—underscores a critical lack of uniformity in the standardisation of commercial botanical extracts.

Furthermore, in terms of toxicological safety, the concentrations of cadmium and lead in nearly all analysed supplements remained remarkably low, falling well within the strict thresholds established by the regulations of the European Union. Nevertheless, the fact that sample H1 marginally exceeded the statutory limit for cadmium and sample B2 closely approached it highlights the inherent vulnerability of hyperaccumulating plant species to environmental pollution. Ultimately, these findings emphasise that continuous independent quality control monitoring of over-the-counter herbal formulations is imperative to ensure consumer safety and prevent chronic exposure to heavy metals.

## Figures and Tables

**Table 1:** Optimised experimental conditions for the determination of Cd, Cu, Pb, and Si using HR-CS GFAAS.

**General Instrumental Settings**	**Cd**	**Cu**	**Pb**	**Si**
Wavelength (nm)	228.802	324.754	283.306	251.611
Lamp current (A)	9			
Spectral range (pixel)	200			
Dispersion (pm pixel^−1^)	2			
Read time (s)	5			
Delay time (s)	0			
Measurement mode	peak height			
Sample volume (μL)	20			
Modifier solution (mixture)	Pd(NO_3_)_2_/Mg(NO_3_)_2_			
Modifier concentration (μg μL^−1^)	1.5/1			
Modifier volume (μL)	5			
**Furnace program steps**				
Drying	80 °C, ramp 6 °C s^−1^, hold 20 s			
Drying	90 °C, ramp 3 °C s^−1^, hold 20 s			
Drying	110 °C, ramp 5 °C s^−1^, hold 10 s			
Pyrolysis	350 °C, ramp 50 °C s^−1^, hold 20 s			
Pyrolysis	600 °C	1100 °C	800 °C	1600 °C
	ramp 300 °C s^−1^, hold 10 s			
Atomisation	1400 °C	2500 °C	1900 °C	2600 °C
	ramp 1500 °C s^−1^, hold 4 s			
Cleanout	2450 °C	2550 °C	2450 °C	2650 °C
	ramp 500 °C s^−1^, hold 4 s			

**Table 2:** Determination of Cd, Cu, Pb, and Si (concentration in mg kg^−1^) in the certified reference material INCT-MP4-2 (Mixed Polish Herbs) using the HRCS GFAAS technique.

Element	Certified Value	Found	Value of *t*-Test	Significance ^3^	Recovery (%)
Cd	0.20 ± 0.02	0.21 ± 0.02 ^1^	1.118	NS	105
Cu	7.77 ± 0.53	7.48 ± 0.35 ^1^	1.853	NS	96
Pb	2.16 ± 0.23	2.08 ± 0.21 ^1^	0.852	NS	104
Si	15 ^2^	14.58 ± 0.81 ^1^	1.159	NS	97

^1^ Standard deviation for five replicate measurements. ^2^ Added (µg L^−1^). ^3^ Significance of the *t*-test (*n* = 5) at 95% confidence level; tcritical = 2.776; NS: not significant.

**Table 3:** Determination of Cd, Cu, Pb, and Si (concentration in µg L^−1^) in dietary supplements following a standard addition using the HR-CS GFAAS technique.

Element	Added	Found	Value of *t*-Test	Significance ^2^	Recovery (%)
Sample B1					
Cd	0.5	0.46 ± 0.04 ^1^	2.236	NS	92
Cu	2.5	2.7 ± 0.3 ^1^	1.491	NS	108
Pb	5.0	5.3 ± 0.2 ^1^	2.236	NS	106
Si	15.0	14.6 ± 0.8 ^1^	1.118	NS	97
Sample B2					
Cd	0.5	0.48 ± 0.03 ^1^	1.491	NS	96
Cu	2.5	2.2 ± 0.4 ^1^	1.677	NS	88
Pb	5.0	4.9 ± 0.2 ^1^	1.118	NS	98
Si	15.0	15.1 ± 0.3 ^1^	0.745	NS	101
Sample B3					
Cd	0.5	0.51 ± 0.03 ^1^	0.745	NS	102
Cu	2.5	2.4 ± 0.2 ^1^	1.118	NS	96
Pb	5.0	5.5 ± 0.5 ^1^	2.236	NS	110
Si	15.0	14.6 ± 0.6 ^1^	1.491	NS	97
Sample H1					
Cd	0.5	0.47 ± 0.03 ^1^	2.236	NS	94
Cu	2.5	2.6 ± 0.1 ^1^	1.118	NS	104
Pb	5.0	4.9 ± 0.2 ^1^	1.118	NS	98
Si	15.0	15.3 ± 0.7 ^1^	0.958	NS	102
Sample H2					
Cd	0.5	0.52 ± 0.03 ^1^	1.491	NS	104
Cu	2.5	2.3 ± 0.3 ^1^	1.491	NS	92
Pb	5.0	4.8 ± 0.3 ^1^	1.491	NS	96
Si	15.0	14.8 ± 0.8 ^1^	0.559	NS	99
Sample H3					
Cd	0.5	0.53 ± 0.04 ^1^	1.677	NS	106
Cu	2.5	2.6 ± 0.2 ^1^	1.118	NS	104
Pb	5.0	4.7 ± 0.4 ^1^	1.677	NS	94
Si	15.0	15.4 ± 0.7 ^1^	1.278	NS	103
Sample N1					
Cd	0.5	0.49 ± 0.02 ^1^	1.118	NS	98
Cu	2.5	2.8 ± 0.3 ^1^	2.236	NS	112
Pb	5.0	5.2 ± 0.3 ^1^	1.491	NS	104
Si	15.0	15.2 ± 0.7 ^1^	0.639	NS	101
Sample N2					
Cd	0.5	0.47 ± 0.04 ^1^	1.677	NS	94
Cu	2.5	2.3 ± 0.3 ^1^	1.491	NS	92
Pb	5.0	5.3 ± 0.3 ^1^	2.236	NS	106
Si	15.0	14.3 ± 0.7 ^1^	2.236	NS	95
Sample N3					
Cd	0.5	0.53 ± 0.03 ^1^	2.236	NS	106
Cu	2.5	2.8 ± 0.3 ^1^	2.236	NS	112
Pb	5.0	5.1 ± 0.3 ^1^	0.745	NS	102
Si	15.0	15.3 ± 0.6 ^1^	1.118	NS	102

^1^ Standard deviation for five replicate measurements. ^2^ Significance of the *t*-test (*n* = 5) at 95% confidence level; t_critical_ = 2.776; NS: not significant.

**Table 4:** Total concentration (C) and mass content per unit (m) of Cd, Cu, Pb, and Si in the analyzed dietary supplements determined by HR-CS GFAAS.

Sample	Parameter	Cd	Cu	Pb	Si
B1	C ^1^	0.161 ± 0.006	162.2 ± 5.7	0.142 ± 0.008	4727.1 ± 18.9
	m ^2^	0.177 ± 0.009	179.7 ± 2.1	0.156 ± 0.006	5231 ± 207
B2	C ^1^	0.823 ± 0.029	188.5 ± 9.4	0.052 ± 0.003	392.1 ± 13.7
	m ^2^	0.933 ± 0.048	213.3 ± 3.6	0.060 ± 0.003	4449 ± 150
B3	C ^1^	0.198 ± 0.010	527.1 ± 18.4	0.068 ± 0.003	355.6 ± 14.2
	m ^2^	0.231 ± 0.012	505.3 ± 21.2	0.078 ± 0.003	4150 ± 165
H1	C ^1^	1.122 ± 0.045	2.1 ± 0.1	0.042 ± 0.002	224.9 ± 8.9
	m ^2^	1.320 ± 0.060	2.4 ± 0.3	0.048 ± 0.007	264.9 ± 10.5
H2	C ^1^	0.131 ± 0.007	550.3 ± 22.2	0.054 ± 0.003	273.1 ± 10.9
	m ^2^	0.151 ± 0.009	514.4 ± 25.8	0.063 ± 0.006	319.5 ± 12.6
H3	C ^1^	0.138 ± 0.007	0.212 ± 0.011	0.044 ± 0.002	182.1 ± 7.3
	m ^2^	0.156 ± 0.009	0.24 ± 0.03	0.049 ± 0.007	204.0 ± 8.1
N1	C ^1^	0.147 ± 0.005	0.169 ± 0.009	0.059 ± 0.002	126.3 ± 5.0
	m ^2^	0.165 ± 0.006	0.18 ± 0.03	0.066 ± 0.003	143.1 ± 5.7
N2	C ^1^	0.151 ± 0.008	0.197 ± 0.011	0.047 ± 0.002	112.7 ± 4.5
	m ^2^	0.158 ± 0.011	0.21 ± 0.03	0.051 ± 0.003	131.4 ± 5.1
N3	C ^1^	0.138 ± 0.007	0.121 ± 0.012	0.049 ± 0.003	98.4 ± 2.9
	m ^2^	0.151 ± 0.010	0.15 ± 0.03	0.054 ± 0.002	109.2 ± 3.3

^1^ Concentration in µg g^−1^ ± SD (*n* = 5). ^2^ Content per tablet or capsule in µg ± SD (*n* = 5).

## Data Availability

The original contributions presented in this study are included in the article. Further inquiries can be directed to the corresponding author.
